# Transcranial Direct Current Stimulation (tDCS) over the Left Dorsal Lateral Prefrontal Cortex in Children with Autism Spectrum Disorder (ASD)

**DOI:** 10.1155/2021/6627507

**Published:** 2021-06-19

**Authors:** Jiujun Qiu, Xuejun Kong, Jihan Li, Jie Yang, Yiting Huang, Minshi Huang, Binbin Sun, Jiayi Su, Helen Chen, Guobin Wan, Jian Kong

**Affiliations:** ^1^Department of Child Psychiatry and Rehabilitation, Affiliated Shenzhen Maternity & Child Healthcare Hospital, Southern Medical University, Shenzhen, China; ^2^MGH/HST Martinos Center for Biomedical Imaging, Massachusetts General Hospital, Harvard Medical School, 149 13th Street, 1118A, Charlestown, MA 02129, USA; ^3^Department of Psychiatry, Massachusetts General Hospital, Harvard Medical School, Charlestown, MA 02129, USA

## Abstract

Recently, transcranial direct current stimulation (tDCS) has been applied to relieve symptoms in individuals with autism spectrum disorder (ASD). In this prospective, parallel, single-blinded, randomized study, we investigate the modulation effect of three-week tDCS treatment at the left dorsal lateral prefrontal cortex (DLPFC) in children with ASD. 47 children with ASD were enrolled, and 40 (20 in each group) completed the study. The primary outcomes are Childhood Autism Rating Scale (CARS), Aberrant Behavior Checklist (ABC), and the Repetitive Behavior Scale-Revised (RBS-R). We found that children with ASD can tolerate three-week tDCS treatment with no serious adverse events detected. A within-group comparison showed that real tDCS, but not sham tDCS, can significantly reduce the scores of CARS, Children's Sleep Habits Questionnaire (CSHQ), and general impressions in CARS (15th item). Real tDCS produced significant score reduction in the CSHQ and in CARS general impressions when compared to the effects of sham tDCS. The pilot study suggests that three-week left DLPFC tDCS is well-tolerated and may hold potential in relieving some symptoms in children with ASD.

## 1. Introduction

Autism Spectrum Disorder (ASD) is a common neurodevelopmental disorder with no cure. Multiple interventions such as behavioral analysis (ABA), occupational therapy, speech therapy, physical therapy, and pharmacological therapy have been used to relieve the symptoms of ASD [1, 2]. Nevertheless, most of these treatments have achieved only limited success. Many (but not all) individuals with ASD require lifelong support of some kind [3]. Thus, there is an urgent need to develop new ASD interventions.

Transcranial direct current stimulation (tDCS) is a safe, low-cost neuromodulation tool that can noninvasively change cortical excitability by applying a low direct current (usually no more than 2 mA) from electrodes placed on the scalp [4–7]. Accumulating evidence suggests that tDCS application to a certain brain region, such as the frontal cortex, can significantly modulate attention [8], learning [8–10], memory [9, 11], vigilance [12], brain activity/connectivity/plasticity/dynamics [13–17], conditioning/placebo effect [18, 19], and neurotransmitter levels ([13]). These unique characteristics make tDCS a promising tool for ASD treatment.

Recently, tDCS has been applied to relieve symptoms in individuals with ASD [20–23]. One widely used target brain region of tDCS is the dorsal lateral prefrontal cortex (DLPFC). As a key region of executive functions, the DLPFC plays an important role in cognitive control processes [24–29]. Cognitive control includes a set of brain processes necessary for goal-directed thought and action [30], which plays a crucial role in the top-down modulation of attention-memory interactions [31, 32], decision-making, and conflict resolution [32].

In parallel, studies have shown that plateaued growth of attentional control is associated with elevated autism trait characteristics and lower adaptive functioning at earlier stages of development [33]. Meta-analysis has also shown that ASD is associated with DLPFC structure and functional alternation [34, 35], which illustrates the important role of the DLPFC in the pathophysiology of ASD. For instance, in a previous study, investigators found that during a parametrically modulated vigilance task, both ADHD and ASD disorders displayed shared under-activation relative to the healthy controls in the bilateral striato-thalamic and the left DLPFC. In addition, the left DLPFC under activation was positively associated with the prosocial Strength and Difficulty Questionnaire (SDQ) scores. This finding may suggest that problems with reducing self-referential thoughts that interfere with frontal attention networks may be either a cause or a consequence of abnormalities in reciprocal social communication [36].

Furthermore, the DLPFC is also the highest cortical area that is involved in motor planning, organization, and regulation/inhibition and has closely connected to other regions such as the orbitofrontal cortex, thalamus, parts of the basal ganglia (specifically, the dorsal caudate nucleus), the hippocampus, and primary and secondary association areas of the neocortex (including posterior temporal, parietal, and occipital areas). This function and connection also linked the DLPFC with behavioral abnormality, such as restricted and repetitive behaviors, hypersensitivities (over-responsiveness), and hyposensitivities (under-responsiveness), to a wide range of stimuli. As a result, it has been used as a target brain region of tDCS for the treatment of ASD.

In an early pilot study [37], researchers tested the hypothesis that tDCS can facilitate language acquisition in a cohort (*n* = 10) of ASD children with immature syntax using a single arm design (pre-tDCS vs. post-tDCS). The anodal lead was placed over the left DLPFC corresponding to F3 of 10-20 EEG system. The cathode was placed over the right supraorbital region (0.08 mA, 30 minute). They found a large effect size of the difference between the pre-/post-tDCS groups on language (syntax) acquisition. In another earlier study [38], investigators explored the effects of tDCS on the left DLPFC using a crossover design on 20 children with ASD. They found that 5 consecutive days of real tDCS treatment (1 mA, 20 minutes, with the anodal electrode placed over F3 and the cathodal electrode on the right shoulder contralateral to the anode) produced a significant decrease in Childhood Autism Rating Scale (CARS) score and Autism Treatment Evaluation Checklist (ATEC) score and produced an increase in the Children's Global Assessment Scale (CGAS) score. In a following study [39] by the same group, the authors studied the effects of anodal tDCS on Peak EEG Alpha Frequency (PAF). Twenty male children on the autism spectrum were randomly assigned in a crossover design to receive a single session of both active and sham tDCS stimulations over the left DLPFC. The investigators found clinical improvement similar to a previous study, as well as PAF increase. In a case report [40], investigators found that 28 consecutive daily tDCS sessions (excluding the weekend, the cathode over the right dorsolateral prefrontal cortex and the anode over the left DLPFC, 1 mA, 20 min) can result in a reduction of catatonic symptoms in an adolescent with ASD and drug-resistant Catatonia.

More recently, investigators applied a crossover design to investigate the modulation of tDCS on working memory in adults with high-functioning autism. Each participant received left anodal/right cathodal stimulation, right anodal/left cathodal stimulation, or sham stimulation, in a randomized, counterbalanced order on three separate days. They found that a single tDCS treatment (40 min of 1.5 mA), through both left DLPFC anodal stimulation and right DLPFC anodal stimulation, can significantly improve working memory task performance and may reduce some core symptoms of ASD [41].

Most recently, Hadoush et al. [42] studied the potential therapeutic effects of tDCS on the clinical characteristics of children with ASD. The tDCS treatment group underwent 10 sessions (1 mA, 20 min durations, five per week) of bilateral anodal tDCS stimulation applied simultaneously over the left and right prefrontal and motor areas (FC1 and FC2 of EEG system) and the cathode electrodes applied over the left and right supraorbital areas. The control group underwent the same procedures but with the use of sham tDCS stimulation. The results showed that there were significant decreases in total autism treatment evaluation checklist (ATEC) scores (*p* = 0.014), sociability subscores (*p* = 0.021), and behavioral, health, and physical condition subscores (*p* = 0.011) in the tDCS treatment group. No significant changes were observed in total ATEC scores and subscores in the control group, demonstrating the potential of tDCS.

Nevertheless, most clinical studies have only tested the short-term (1 to 5 treatments) effects of tDCS. Although crossover design can increase the power of the study, the “carry-over” effect of tDCS may confound estimates of the effect of tDCS treatment. We thus performed a pilot prospective, single-blinded, randomized, parallel clinical study to test the efficacy of three-week tDCS at the left DLPFC [38, 39] in children with ASD. We hypothesized that three weeks of tDCS treatment can be well-tolerable and may significantly reduce the symptoms associated with ASD children.

## 2. Materials and Methods

### 2.1. Participants

Participants in the ASD group were recruited from an outpatient ward of the Child Mental Health and Rehabilitation Center in the Shenzhen Maternity & Child Healthcare Hospital in China. The clinical trial registration number is ChiCTR1800015264. Written informed consent to participate in this study was provided and signed by the participants' legal guardians before the start of the experiment.

The inclusion criteria were that the participant had to be 2-6 years old and had to meet the diagnostic criteria for DSM-V autism spectrum disorders as confirmed by study physicians. The exclusion criteria were the use of neurologic, hormone, or immunological therapy within three months; mental illness (schizophrenia, etc.), hereditary metabolic diseases, severe neurological diseases, and craniocerebral injury history; history of epilepsy; and intracranial implants or other conditions that lead to the inability to administer tDCS treatment. All children received regular special education provided by special schools for ASD.

### 2.2. Experimental Procedure

#### 2.2.1. Randomization and Blindness

After screening, eligible children were randomly assigned in a 1 : 1 ratio to the real or sham tDCS groups using computer-generated randomized numbers. All guardians/children and investigators/staffs, except a staff who applied the tDCS, were blinded to real or sham tDCS interventions.

#### 2.2.2. Transcranial Direct Current Stimulation (tDCS) Administration

The tDCS treatment will be administered using two saline-soaked, surface sponge electrodes (area: 5 × 5 cm^2^) and delivered using the Brain Stimulator v3.0 tDCS device (https://www.amazon.ca/Brain-Stimulator-v3-0-Device-Deluxe/dp/B01N74RKEI) by trained research clinicians under the supervision of the study physician and aid of parents. All devices were placed in a carrying pouch provided by the device vendor and tied to the back of the waist belt to blind the patients and parents. tDCS was applied five times a week for three weeks.

Similar to previous studies [38, 39] that showed significant clinical improvement after tDCS in children with ASD, for real tDCS, the anodal electrode was placed over F3 of a 10-20 EEG electrode placement (the site of the left dorsal lateral prefrontal cortex) and the cathodal electrode was placed on the right shoulder contralateral to the anode. tDCS treatment was applied at 1 mA for twenty minutes. For sham tDCS after the setup electrodes, the staff turned on the device for 15 seconds, then turned it off and placed it in the carrying pouch. Thus, no stimulation was applied except at the beginning of the sham stimulation to mimic the somatosensory effect of real tDCS for 15 seconds.

### 2.3. Clinical Outcome

The primary outcomes include three commonly used measurements that reflect key symptoms associated with ASD, i.e., Childhood Autism Rating Scale (CARS, higher scores indicate greater severity), Aberrant Behavior Checklist (ABC, higher scores indicate greater severity), and Repetitive Behavior Scale-Revised (RBS-R, higher scores indicate greater severity). We chose CARS because it is a well-established measure of autism severity [43], and previous studies have found that tDCS can reduce the CARS score [38]. We chose ABC because it is one of the few empirically developed scales designed to measure psychiatric symptoms and behavioral disturbance exhibited by individuals with developmental disabilities [44]. The ABC is a caregiver-informed problem behavior scale that assesses five categories: Irritability, Agitation, and Crying; Lethargy/Social Withdrawal; Stereotypic Behavior; Hyperactivity/Noncompliance; and Inappropriate Speech. It is a widely used measure in ASD treatment studies [45]. Repetitive and stereotyped patterns of behavior, interests, and activities have been considered central to ASD[46]; we thus also included RBS-R as a primary outcome. RBS-R is a self-reported questionnaire that is used to measure the breadth of repetitive behavior for ASD individuals. It consists of 6 subcategories: stereotyped behavior, self-injurious behavior, compulsive behavior, routine behavior, sameness behavior, and restricted behavior for a total score of 43 items.

The secondary outcomes are sleep condition, as measured by the Children's Sleep Habits Questionnaire (CSHQ, higher scores indicating more severe sleep disturbance), the subscale scores of ABC and RBS-R, and the 15 items (questions) of CARS. For all secondary outcomes, higher scores indicated greater severity. We include the general impressions (the 15^th^ item in CARS) as a separate secondary outcome, as it represents an overall rating of autism based on subjective impression of the degree to which the child is autistic given all of the available information [47]. All outcome measurements (when applicable) were applied by trained clinicians who were blinded to the treatment group at baseline and within two weeks after the final tDCS treatment.

#### 2.3.1. Adverse Events

Similar to a previous study [38], clinicians and the parents/guardians were asked to report any adverse events as well as other signs and symptoms every day after treatment. Participants were also closely observed by clinicians during the stimulation session.

### 2.4. Statistical Analysis

The effect of tDCS was estimated by comparing primary outcome differences using a mixed-model regression with group (real vs. sham) and time points (baseline and after treatment) as fixed effects and with the patients as a random effect. Age and gender were also included in the model as covariates. All analyses were performed using R Version 3.1.0 with the lme4 (http://CRAN.R-project.org/package=lme4) and lmerTest packages (http://CRAN.R-project.org/package=lmerTest). For this model, the treatment effect is the group-by-time interaction. Within-group comparisons before and after treatment were performed using paired *t*-tests. We also performed the same analyses on the secondary clinical outcomes.

### 2.5. Power Calculation

Since no prior clinical trial has been performed to investigate the effects of three-week tDCS on the left DLPFC, we present our power analysis here for the primary outcomes before and after three-week tDCS treatments. For the comparison of the pre- and posttreatment differences between the real and sham tDCS groups, with 20 children on the autism spectrum in each group, we will use 80% power to test the effect size of 0.91 (Cohen-*d*) between the two groups, based on the two sample *t*-tests at the 0.05 two-tailed significance level.

## 3. Results

### 3.1. Participants and Baseline Characteristics

**Figure 1:**
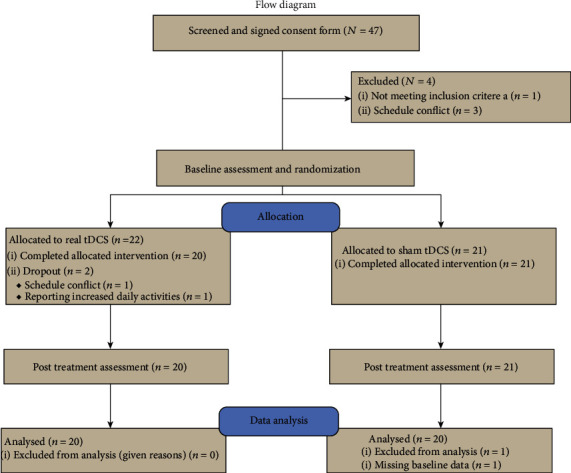
Flow chart of the experiment.

47 guardians of the participants signed the consent form and were enrolled in this study. Four subjects dropped at the baseline assessment stage before randomization (three due to schedule conflicts and one due to noncompliance with the inclusion criteria). 43 participants were randomized to real (*n* = 22) or sham (*n* = 21) tDCS treatment. Two subjects dropped from the real tDCS group (one due to a schedule conflict and one due to a mother's concern of an increase in daily activity/movement after treatments). One subject's data in the sham tDCS group was not used due to missing data at the baseline (Figure 1).

Of the 40 subjects who completed the study, the ABC, RSB, and CSHQ of three subjects (all in the sham group) could not be assessed by the same person (guardian) before and after treatments; thus, the final data analyses for ABC, RSB, and CSHQ were performed on 37 subjects (20 in the real tDCS group and 17 in the sham tDCS group). The final data analysis for CARS, which was assessed by trained staff, was performed on 40 subjects (20 in each group) who completed the study.

The baseline demographic characteristics are shown in Table 1. There are no significant differences in age (*p* = 0.98) and gender (*p* = 0.53) and baseline clinical assessments between the real and sham tDCS groups (*p* value range from 0.12 to 0.97).

### 3.2. Primary Outcome Results

**Figure 2:**
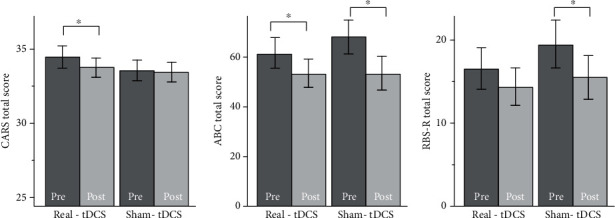
Effects of real and sham tDCS on Childhood Autism Rating Scale (CARS), Aberrant Behavior Checklist (ABC), and Repeat Behavior Scale - Revised (RBS-R). ∗ indicates a significant difference before and after treatment (within group comparison, *p* < 0.05).

Within-group data analysis (paired *t*-test) on primary outcomes showed that after real tDCS treatment, ABC and CARS score significantly decreased (improvement) (*t*(19) = 2.2, *p* = 0.04; *t*(19) = 2.1, *p* = 0.05, respectively). After sham tDCS, ABC score and RBS-R score were reduced (improvement) significantly (*t*(16) = 4.0, *p* < 0.001; *t*(16) = 3.0, *p* = 0.01, respectively) (Figure 2).

Comparison between the two groups using mixed-model regression analysis showed no significant difference in group-by-time interaction for ABC total score (*p* = 0.21), RBS-R (*p* = 0.37), and CARS score (*p* = 0.42).

We also explored the association (using Pearson's correlation) between the baseline and pre- and posttreatment differences (pre- minus posttreatment) in three primary outcomes (CARS, ABC, and RBS-R) that showed significant changes after real or sham tDCS treatment during within group analysis. We found significant correlation between baseline CARS score and CARS score decrease (improvement) after real tDCS (*p* = 0.03, *r* = 0.49), baseline ABC score, and ABC score change after real tDCS (*p* = 0.04, *r* = 0.46). There is no significant association between the baseline ABC/RBS-R score and ABC/RBS-R score change after sham tDCS.

### 3.3. Secondary Outcome Results

Within-group data analysis (paired *t*-test) on secondary outcomes showed that after real tDCS treatment, ABC stereotypic behavior subscale score (*t*(19) = 2.2, *p* = 0.04), and ABC hyperactivity subscale score (*t*(19) = 2.8, *p* = 0.01), and CSHQ score (*t*(19) = 2.6, *p* = 0.02) were significantly reduced (improvement) (Table 2).

After sham tDCS, ABC subscale irritability (*t*(16) = 3.2, *p* = 0.01), ABC social subscale withdrawal (*t*(16) = 3.5, *p* = 0.003), ABC stereotypic behavior subscale score (*t*(16) = 2.5, *p* = 0.02), ABC hyperactivity subscale score (*t*(16) = 3.2, *p* = 0.006), and RBS-R ritualistic/sameness subscale score (*t*(16) = 2.3, *p* = 0.04) were significantly reduced (improvement). No significant increase was detected after the treatments in all secondary outcomes.

Between-group comparisons showed significant differences (group-by-time interaction) between the two groups on CSHQ score (*p* = 0.049, real tDCS produced a greater decrease), the general impression item in CARS (*p* = 0.047, real tDCS produced a greater decrease), and ABC subscale irritability (*p* = 0.01) (sham tDCS produced a greater decrease). No other significant secondary outcome differences were detected (*p* value group-by-time interaction ranging from 0.1 to 0.89).

## 4. Discussion

In this pilot study, we investigated the modulation effect of a 3-week left frontal anodal tDCS treatment on children with ASD. We found no signifiant difference in pre- and posttreatment changes for ABC, RSB, and CARS scores between the real and sham tDCS groups. Nevertheless, we found that after real tDCS treatment, but not after sham tDCS treatment, CARS scores were reduced significantly. Exploratory analysis showed that real tDCS can significantly reduce the CSHQ score and the general impression item in CARS when compared to the effect of sham tDCS. Our results suggest that tDCS may hold the potential in relieving some symptoms of ASD.

We found that real tDCS treatment can significantly decrease CARS and ABC scores. This result is consistent with a previous cross-over study [38] in which the authors found that five-day real tDCS treatment (five-session) can produce a significant decrease in the CARS score. Our finding is also consistent with a noncontrolled study, in which the investigator found that both real tDCS and repetitive Transcranial Magnetic Stimulation at left DLPFC can significantly reduce ABC scores in children with ASD [48].

The CARS is a 15-item rating scale used to identify children on the autism spectrum and distinguish them from those with developmental disabilities [43]. In addition, it is a well-established autism severity measurement. Unlike the other two primary outcomes (ABC and RSB), which were assessed by the guardians of the children with ASD, CARS was measured by trained clinicians who were blinded to the treatment modes. Thus, it may be associated with less bias than the other two primary outcomes (ABC and RSB).

Interestingly, we found significant correlation between baseline CARS/ABC scores and CARS/ABC score changes (pre- minus post-treatment) after real tDCS. These results may indicate that those with more severe symptoms tend to have a greater response to tDCS treatment. This is consistent with previous studies showing that baseline severity level can be used as a predictor of treatment response [49, 50].

We did not detect a significant CARS total score difference between the two groups; this may be due to the small sample size in this pilot study. Nevertheless, we detected a significant difference in the general impression (item 15 of CARS) between the two groups. Real tDCS produced a small but significant general impression score decrease (improvement) compared to the effect of sham tDCS. The general impressions item (the 15^th^ item in CARS) is an overall rating of autism based on subjective impression of the degree to which the child is autistic, as defined by the other 14 items of CARS. This rating is not an average rating of the other 14 items. It was made based on all of the available information including the case history, parent interview, and past records [47]. Thus, it represents a summarized overview of the general conditions. The significant improvement in general impression scores further endorsed the potential of tDCS. Future studies with larger sample sizes are needed to replicate this finding.

We also found that the CSHQ score was reduced significantly after real tDCS treatment and that there were significant differences in CSHQ score changes after treatment between the real and sham tDCS groups with a medium effect size (Cohen's *d* = 0.66). This result implies that real tDCS can also improve sleep quality in children with ASD.

The CSHQ is a retrospective parent questionnaire that has been used to examine sleep behavior in young children [51–53]. The literature suggests that children and adults with autistic symptoms often experience sleep disturbances and alterations in circadian sleep rhythmicity. Disturbed sleep is one of the most common reasons that parents with children who have ASD seek medical support [54], with the prevalence rate of sleep disturbances between 64% and 93% [55]. In particular, total sleep time is reduced starting from 30 months of age towards adolescence [55]. Studies further suggest improving treatment strategies for both children and adults with ASD by targeting sleep disturbances [55].

To further support our findings, previous studies found that the prefrontal cortex (including the left DLPFC) plays a role in mediating normal sleep physiology, dreaming, and sleep-deprivation phenomena. Particularly, studies showed that during nonrapid-eye-movement sleep, frontal cortical activity is characterized by the highest voltage and the slowest brain waves when compared to other cortical regions [56–58].

Interestingly, we found that both ABC score and RBS-R score reduced significantly after sham tDCS. Sham tDCS treatment also produced greater ABC irritability subscore reduction compared to real tDCS treatment. There is almost no change after real tDCS in ABC irritability subscore (the score reduced 0.1 point); thus, the significant difference in ABC irritability subscore between real and sham tDCS is mainly derived from reduction after sham tDCS (Table 2). This result indicates that tDCS itself is safe and did not change ABC irritability. Our findings also suggest that the placebo effect produced by sham tDCS is robust, particularly in scores assessed by the guardians/parents such as ABC and RBS-R.

During each visit, we asked the parent/guardian if they detected any unusual behavior/symptoms since the last treatment. The children's responses were also assessed by the clinical staff and parents/guardian during the treatment. No serious adverse events were detected/reported during and after tDCS administration. A parent reported hyperactivity after one week of real tDCS treatment for one child. The symptom disappeared in one week after withdrawal from tDCS treatment. No other adverse events were observed/reported.

The mechanism of tDCS treatment of ASD remains unclear. Recently, EEG has been used to investigate the underlying mechanism of ASD and its treatment. Compared with other brain imaging tools such as fMRI, EEG can be applied relatively easily on children, particularly on those with lower developmental abilities.

For instance, studies have found that children with ASD are associated with less relative alpha and more relative delta compared to those in typically developed children [59, 60]. Further studies showed that peak alpha frequency (PAF) as a marker for ASD depends on age, with an resting state alpha marker of more interest in younger versus older children with ASD [61]. Interestingly, in a previous study on tDCS treatment in the DLPFC [39], investigators studied the effects of anodal tDCS using Peak EEG Alpha Frequency. They found (1) significant pre- to postsession improvement in social and health/behavior domains of ATEC following active tDCS treatment and (2) that PAF increased at the stimulation site. The increase in PAF was significantly associated with improvement in the two domains of ATEC impacted by tDCS.

In a more recent study, researchers applied maximum entropy ratio (MER), a new symbolic analysis approach for the detection of recurrence domains of complex dynamical systems from time series, to investigate the modulation effect of tDCS. They found that the MER value significantly increased after repeated DLPFC tDCS (10 tDCS sessions once every other day, 1 mA, 20 min) compared to that of the waiting list control group [62], suggesting that anodal tDCS over the DLPFC can increase EEG complexity.

Studies have shown that, in addition to modulating the EEG signal, anodal tDCS can reduce the GABA levels as well as resting-state functional coupling compared with sham tDCS, reflecting the preserved neuromodulatory effect of tDCS in older adults [13]. Interestingly, studies have suggested the ASD may be associated with imbalance of neural excitation and inhibition (glutamate (Glu) and *γ*-aminobutyric acid (GABA) balance) [63]. Thus, the tDCS may also relieve the symptom of ASD by modulating the neurotransmitter imbalance.

Finally, the tDCS can significantly modulate the brain activity and connectivity [64, 65]. For instance, Antonenko et al. also found that tDCS can reduce resting-state functional coupling compared with sham tDCS [13]. We also found that repeated tDCS (three consecutive sessions) at the DLPFC can modulate (1) the functional connectivity between the DLPFC and the orbital medial prefrontal cortex [19] and (2) the occurrences and transitions of brain dynamics represented by the fMRI coactivation patterns [17]. The literature has suggested that ASD is associated with altered prefrontal functional connectivity [66, 67]. A future study is needed to directly investigate the linkage between tDCS, brain function, and ASD neuropathology.

It is worth noting that we are still in the infant stage of utilizing tDCS for ASD treatment. There are several limitations in this study. First, as a pilot study, the sample size is relatively small. A future study with a larger sample size is needed. Second, a treatment duration of three weeks is still relatively short; a future study with a longer treatment duration is needed. Third, we only applied assessments at two time points (baseline and posttreatment); a future study with multiple assessments including follow-up will provide us with crucial trajectory information of tDCS treatment such as the minimum duration of tDCS and how long the effects can last. Fourth, we chose the left DLPFC as the target region of tDCS. In a recent study, we combined the meta-analysis and functional connectivity methods and found multiple potential tDCS targets including the DLPFC, medial prefrontal cortex, angular gyrus, inferior frontal gyrus, superior parietal lobe, postcentral gyrus, precentral gyrus, middle temporal gyrus, superior temporal sulcus, lateral occipital cortex, and supplementary motor area. Interestingly, the identified DLPFC is slightly different from the F3 locations applied in our study [34]. Thus, future studies are needed to optimize the DLPFC locations. Moreover, studies applied on other target locations, particularly testing if specific target locations can produce specific symptom reduction in individuals with ASD, are needed in the future. Finally, tDCS is just one of the brain stimulation techniques; other brain stimulation methods such as Transcranial Magnetic Stimulation and Transcranial Alternating Current Stimulation [20–23] should also be considered for future studies.

In summary, we found that three-week tDCS treatment at the left DLPFC is feasible and well-tolerated in children with ASD. No serious adverse events were detected. Real tDCS can significantly reduce the scores of CARS, CSHQ, and general impressions of CARS; real tDCS produced significant score reduction in CSHQ and general impressions in CARS compared to that of sham tDCS. The pilot study suggests that tDCS may be a promising treatment option for relieving symptoms in children with ASD.

## Figures and Tables

**Table 1:** Demographic and other related information for the subjects who completed the study.

ID	Group	Gender	Age (months)	Handedness	Parturition
1	Sham tDCS	Female	78	Right	Natural
2	Real tDCS	Male	30	Left	C-section
3	Real tDCS	Male	81	Right	C-section
4	Sham tDCS	Male	54	Left	C-section
5	Sham tDCS	Female	69	Right	Natural
6	Real tDCS	Male	36	Right	C-section
7	Sham tDCS	Male	47	Right	Natural
8	Real tDCS	Male	40	Right	Natural
9	Real tDCS	Female	79	Right	Natural
10	Sham tDCS	Female	70	Right	Natural
11	Real tDCS	Female	42	Right	C-section
12	Sham tDCS	Male	42	Right	Natural
13	Real tDCS	Male	67	Right	C-section
14	Real tDCS	Male	47	Right	Natural
15	Sham tDCS	Female	53	Right	Natural
16	Real tDCS	Male	44	Right	C-section
17	Sham tDCS	Male	44	Right	C-section
18	Real tDCS	Female	59	Right	Natural
19	Sham tDCS	Male	61	Right	C-section
20	Sham tDCS	Male	34	Right	Natural
21	Real tDCS	Male	30	Right	Natural
22	Real tDCS	Male	59	Right	Natural
23	Sham tDCS	Male	50	Right	Natural
24	Sham tDCS	Male	71	Right	C-section
25	Real tDCS	Male	37	Right	Natural
26	Real tDCS	Male	50	Right	Natural
27	Sham tDCS	Female	49	Right	Natural
28	Sham tDCS	Female	24	Right	C-section
29	Real tDCS	Male	65	Right	C-section
30	Sham tDCS	Male	25	Right	Natural
31	Real tDCS	Male	63	Right	Natural
32	Real tDCS	Male	46	Right	Natural
33	Sham tDCS	Male	73	Right	C-section
34	Sham tDCS	Male	44	Right	Natural
35	Real tDCS	Male	54	Right	Natural
36	Real tDCS	Female	62	Right	Natural
37	Sham tDCS	Male	39	Right	C-section
38	Sham tDCS	Male	66	Right	Natural
39	Real tDCS	Male	54	Right	Natural
40	Sham tDCS	Male	49	Right	C-section

**Table 2:** Clinical assessments for each group before and after treatment (mean ± SD).

Group	Real tDCS			Sham tDCS		
	Pretreatment	Posttreatment	Difference (pre-post)	Pretreatment	Posttreatment	Difference (pre-post)
CARS total score	34.5 ± 3.4	33.8 ± 3.0	0.7 ± 1.5^∗^	33.6 ± 3.2	33.5 ± 3.0	0.1 ± 2.4
ABC total score	61.6 ± 27.6	53.5 ± 24.9	8.1 ± 16.5^∗^	68.4 ± 26.8	53.5 ± 27.3	14.9 ± 15.5^∗^
RBS-R total score	16.5 ± 11.2	14.4 ± 10.2	2.1 ± 7.2	19.5 ± 11.7	15.4 ± 10.7	4.1 ± 5.6^∗^
ABC-irritability^**+**^	10.8 ± 6.1	10.9 ± 6.2	−0.1 ± 3.5	14.1 ± 6.5	10.2 ± 5.5	3.9 ± 5.1^∗^
ABC-social withdrawal	19.6 ± 10.1	17.0 ± 9.7	2.7 ± 7.0	17.3 ± 5.9	13.0 ± 6.5	4.3 ± 5.0^∗^
ABC-stereotypic behavior	6.3 ± 4.4	5.2 ± 4.5	1.1 ± 2.2^∗^	6.7 ± 5.5	5.1 ± 4.4	1.6 ± 2.7^∗^
ABC-hyperactivity	20.9 ± 7.5	17.3 ± 7.2	3.6 ± 5.7^∗^	25.8 ± 9.9	21.5 ± 10.7	4.3 ± 5.5^∗^
ABC-inappropriate	4.0 ± 2.5	3.2 ± 2.1	0.8 ± 2.3	4.5 ± 2.9	3.8 ± 2.9	0.7 ± 2.0
RBS-R-stereotyped	3.4 ± 2.9	3.1 ± 2.6	0.3 ± 2.2	4.5 ± 3.3	3.8 ± 2.4	0.6 ± 2.8
RBS-R-self-injurious	0.7 ± 1.2	0.4 ± 0.8	0.3 ± 0.7	0.9 ± 1.3	1.0 ± 2.0	−0.1 ± 1.4
RBS-R-compulsive	2.8 ± 2.9	2.8 ± 3.4	−0.1 ± 1.4	3.0 ± 2.6	2.1 ± 2.7	0.9 ± 2.0
RBS-R-routine	5.0 ± 3.7	4.3 ± 3.3	0.7 ± 2.5	4.1 ± 3.2	3.7 ± 3.1	0.4 ± 2.2
RBS-R-sameness	2.4 ± 2.7	2.0 ± 2.2	0.4 ± 2.0	4.3 ± 3.9	2.9 ± 2.7	1.4 ± 2.4^∗^
RBS-R-restricted	2.4 ± 2.6	1.8 ± 2.4	0.6 ± 2.2	2.8 ± 2.8	1.8 ± 2.1	0.9 ± 2.0
CSHQ^**+**^	53.4 ± 4.9	50.8 ± 4.2	2.7 ± 4.6^∗^	52.5 ± 3.9	53.7 ± 5.9	−1.2 ± 6.8
CARS-relationship to people	2.4 ± 0.4	2.3 ± 0.4	0.1 ± 0.3	2.3 ± 0.4	2.2 ± 0.3	0.1 ± 0.3
CARS-imitation	2.2 ± 0.4	2.1 ± 0.4	0.1 ± 0.3	2.1 ± 0.4	2.1 ± 0.3	0.0 ± 0.3
CARS-emotion response	2.4 ± 0.4	2.3 ± 0.3	0.1 ± 0.2	2.3 ± 0.3	2.2 ± 0.3	0.1 ± 0.4
CARS-body use	2.3 ± 0.4	2.2 ± 0.4	0.1 ± 0.3	2.2 ± 0.3	2.2 ± 0.4	0.0 ± 0.3
CARS-object use	2.4 ± 0.4	2.3 ± 0.4	0.1 ± 0.3	2.2 ± 0.4	2.2 ± 0.4	0.0 ± 0.5
CARS-adaptation to use	2.2 ± 0.3	2.2 ± 0.3	0.0 ± 0.4	2.2 ± 0.4	2.1 ± 0.3	0.1 ± 0.4
CARS-visual response	2.3 ± 0.4	2.2 ± 0.3	0.1 ± 0.4	2.2 ± 0.5	2.2 ± 0.4	−0.1 ± 0.4
CARS-listening response	2.2 ± 0.3	2.3 ± 0.3	0.0 ± 0.3	2.3 ± 0.4	2.2 ± 0.3	0.1 ± 0.4
CARS-taste, small, touch, response and use	2.2 ± 0.3	2.2 ± 0.4	0.1 ± 0.2	2.2 ± 0.4	2.2 ± 0.3	−0.1 ± 0.4
CARS-fear and nervousness	2.2 ± 0.3	2.2 ± 0.3	−0.1 ± 0.4	2.2 ± 0.4	2.2 ± 0.3	0.0 ± 0.3
CARS-verbal communication	2.6 ± 0.2	2.6 ± 0.2	0.0 ± 0.3	2.5 ± 0.3	2.4 ± 0.4	0.1 ± 0.3
CARS-nonverbal communication	2.2 ± 0.2	2.2 ± 0.3	0.0 ± 0.3	2.2 ± 0.3	2.1 ± 0.3	0.1 ± 0.3
CARS-activity level	2.1 ± 0.4	2.0 ± 0.6	0.2 ± 0.6	2.2 ± 0.4	2.3 ± 0.3	−0.1 ± 0.4
CARS-level and consistency of intellectual response	2.4 ± 0.3	2.4 ± 0.3	−0.1 ± 0.2	2.3 ± 0.6	2.4 ± 0.3	−0.1 ± 0.6
CARS-general impressions^**+**^	2.6 ± 0.4	2.5 ± 0.3	0.1 ± 0.3	2.4 ± 0.4	2.5 ± 0.3	−0.1 ± 0.4

For CARS and CARS 15 separate items, there are 20 subjects in each group. For ABC, RBS-R, and CSHQ score and subscores, there are 20 subjects in the real tDCS group and 17 subjects in the sham tDCS group. ∗ indicates a significant difference before and after treatment (within group, *p* < 0.05); **+** indicates a significant difference on pre- and posttreatment changes between the real and sham tDCS groups (between group, *p* < 0.05).

## Data Availability

Data are available upon request.

## Additional Points

*Highlights*. We investigated the effects of 3-week tDCS treatment at the left DLPFC in children with ASD. Children with ASD can tolerate tDCS with no serious adverse event detected. Real tDCS can improve sleep quality and general impressions score when compared to that of sham tDCS.
